# Coronary Artery Ectasia Presenting as a Non-ST Elevation Myocardial Infarction in a Young Adult: Case Presentation and Literature Review

**DOI:** 10.1155/2018/9817812

**Published:** 2018-08-26

**Authors:** Kareem Genena, Mir Ali, Donald Christmas, Henry Siu

**Affiliations:** Department of Medicine, Saint Francis Medical Center, Trenton, NJ 08629, USA

## Abstract

While acute coronary syndromes most commonly occur secondary to unstable atherosclerotic plaque, coronary aneurysms, also known as coronary artery ectasia (CAE), represent a less common etiology. Whereas coronary atherosclerosis accounts for about 50% of CAE, the remaining 50% are either congenital or secondary to a host of inflammatory and connective tissue disorders, with Kawasaki disease being a well-known association. Patients with CAE have worse outcomes than the general population regardless of the presence of associated atherosclerotic coronary artery disease. We report the case of a young male presenting with chest pain, a right bundle branch block on electrocardiography, an elevated troponin level, and a regional wall motion abnormality on echocardiography who is found to have diffuse coronary artery ectasia on coronary angiography and is managed medically with dual antiplatelet therapy.

## 1. Introduction

Coronary artery aneurysms, also known as coronary artery ectasia (CAE), are an uncommon cause of acute coronary syndromes. They are most often secondary to atherosclerotic coronary artery disease but could be congenital and could be the sequela of vasculitic coronary disorders.

## 2. Case Presentation

A 26-year-old African-American gentleman presents to the emergency department with pressure-like retrosternal chest pain that occurred one hour after he completed a workout. His pain subsided after ingestion of an antacid. He has no known medical history and takes no medications. He is a current smoker with a pack-year index of 22. He smokes marijuana but denies other illicit drug use. Both his father and grandfather had hypertension, diabetes, and peripheral arterial disease requiring limb amputation, but there was no known family history of coronary artery disease.

**Figure 1:**
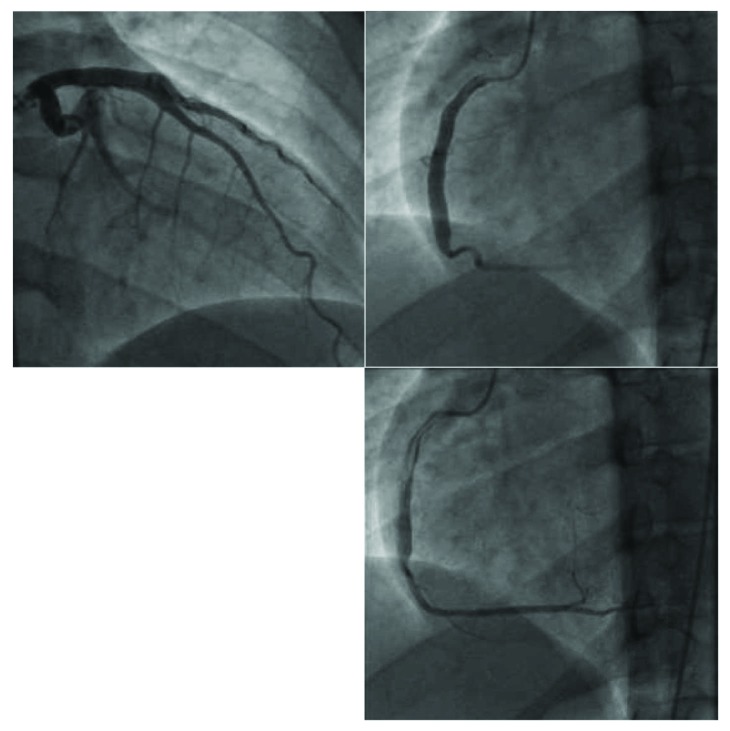
Diffuse coronary artery ectasia in the left coronary system (a) and the right coronary artery (b). Note the large caliber of the proximal vessels in comparison to the distal vessels.

EKG revealed a right bundle branch block with no ST segment or T wave changes indicating ischemia. Initial troponin level was elevated at 8 ng/ml (normal range: <0.03 ng/ml). The patient refused to be admitted for further evaluation and left the hospital against medical advice. He returned a week later, with no symptoms, only to complete the evaluation of his prior episode of chest pain. Echocardiography revealed akinesis in the basal and inferior walls with an ejection fraction of 50%. Coronary angiography revealed moderately to severely dilated aneurysms in the proximal segments of the left anterior descending, left circumflex, and right coronary arteries without flow-limiting lesions (Figure 1).

The patient had no recollection of any febrile childhood illness that would be consistent with childhood Kawasaki disease. He was discharged on dual antiplatelet therapy and a high-intensity statin, as well as metformin for a new diagnosis of prediabetes.

## 3. Discussion

Coronary aneurysms are defined by dilation of a segment of the coronary arteries that is >1.5 times the adjacent normal segment [1]. In one series of 3870 patients undergoing coronary angiography in Italy, the prevalence of CAE was 3.6% [2]. Other angiographic series have reported prevalence rates that varied widely, from 0.3% to 12% [3].

The most common cause of CAE is atherosclerotic coronary disease. However, CAE could be congenital and could be secondary to vasculitic disorders affecting the coronaries such as Kawasaki disease, connective tissue diseases such as Marfan syndrome, or infectious etiologies such as mycotic aneurysms. Coronary aneurysms could also be iatrogenic, after coronary intervention. Atherosclerotic and congenital coronary aneurysms account for 70–80% of all CAE [3].

Reports of the contribution of the traditional risk factors for atherosclerotic coronary artery disease to the formation of coronary aneurysms have varied. In one study of 32,372 patients undergoing coronary angiography in the United States, aneurysmal patients presented similar frequencies of hypertension (61% versus 55%, *p* = 0.13) and a smoking history (47% versus 41%, *p* = 0.08) as did controls. Moreover, patients with aneurysms were less likely to be diabetic (18% versus 26%, *p* = 0.01) and/or current smokers (13% versus 19%, *p* = 0.03). Hyperlipidemia (55% versus 44%, *p* = 0.002) and male gender (83% versus 60%, *p* = 0.0001) were the only traditional risk factors that were identified in a higher frequency in aneurysmal patients in this study [4]. This only points to the fact that CAE and atherosclerotic CAD share common risk factors and does not imply that smoking or hypertension is not a risk factor for CAE. It can be seen in the latter study that 61% and 47% of patients with CAE had hypertension and a smoking history, respectively. Smoking is a known risk factor for abdominal aortic aneurysms. Patients with abdominal aortic aneurysms have an odds ratio of 2.4 of being smokers according to a meta-analysis of 14 cross-sectional studies [5].

Myocardial infarction in patients with CAE could occur due to associated atherosclerotic coronary artery disease and could occur due to aneurysm thrombosis secondary to stagnant nonlaminar flow that is not related to plaque rupture.

Medical therapy for CAE includes antiplatelet agents and guideline-directed medical therapy for associated cardiovascular risk factors such as hypertension, diabetes, and hyperlipidemia. Anticoagulation is suggested for larger caliber aneurysms on the premise of an increased risk of aneurysm thrombosis. Percutaneous intervention is challenging due to large vessel caliber, thrombus burden within the aneurysm, and the potential need for a covered stent. When thrombus is identified within the aneurysm, thrombus aspiration can be attempted. In cases where residual thrombus remains in the artery despite aspiration, some authors advocate for triple antithrombotic therapy, that is, dual antiplatelet therapy and an anticoagulant. Low-dose rivaroxaban (15 mg once daily) has been used by some authors [6]. Solitary bypass grafting is suggested as the intervention of choice for nongiant aneurysms by some authors [7]. Other surgical options include aneurysm ligation, resection, or marsupialization with interposition grafting [3, 7].
